# The efficacy and adverse effects of budesonide in remission induction treatment of autoimmune hepatitis: a retrospective study

**DOI:** 10.3325/cmj.2019.60.345

**Published:** 2019-08

**Authors:** Ömer Burcak Binicier, Süleyman Günay

**Affiliations:** 1 Department of Gastroenterology, Tepecik Training and Research Hospital, İzmir, Turkey; 2 Department of Gastroenterology, İzmir Katip Çelebi University Atatürk Training and Research Hospital, İzmir, Turkey

## Abstract

**Aim:**

To compare the early biochemical response and rate of adverse effects in patients who received prednisolone (PRED)/azathioprine (AZA) and those who received budesonide (BUD)/AZA as the first-line treatment for autoimmune hepatitis.

**Methods:**

The study involved 25 patients receiving PRED 30 mg/day + AZA 50 mg/day and 25 patients receiving BUD 9 mg/day + AZA 50 mg/day from February 2015 to February 2018. Biochemical and hemogram data at baseline and after 6 months of treatment, and adverse effects observed in the follow-up, were compared.

**Results:**

There was no difference between the groups in biochemical response (17 patients receiving PRED/AZA and 18 receiving BUD/AZA) and the rate of adverse effects (9 patients receiving PRED/AZA and 5 receiving BUD/AZA). The total number of adverse effects in the BUD/AZA group was lower (15 vs 7) and the treatment was discontinued in 2 (8%) patients in PRED/AZA group, while no treatment discontinuation was observed in BUD/AZA group.

**Conclusions:**

This study showed no differences in biochemical response between the groups. Lower, although not significantly, rate of adverse effects and lower total number of adverse effects indicate that BUD/AZA may potentially be used as the first-line treatment of choice, especially in patients with obesity, diabetes, resistant hypertension, glaucoma, or osteoporosis.

AIH is an autoimmune liver disease that may lead to chronic liver disease and cirrhosis in untreated patients. The disease is characterized by autoantibody positivity and elevated levels of gamma globulins with necroinflammatory activity and signs of chronic hepatitis, and is often histopathologically associated with interface hepatitis (1,2). Disease affects patients of all ages, predominantly women (women/men:4/1) (3). The diagnosis is based on diagnostic criteria rather than on a specific laboratory test. AIH is classified according to serological test results into type 1 and type 2 disease. Type 1 AIH is characterized by the presence of anti-nuclear antibodies (ANA) and/or anti-smooth muscle antibodies (anti-SMA). Type 2 disease is characterized by the presence of anti-liver kidney microsomal antibodies type 1 (anti-LKM1) and/or anti-liver cytosol (anti-LC1) positivity (1).

AIH is the first liver disease with a demonstrated effect of pharmacological treatment on survival. Response rates with steroid-based immunosuppressive therapies range from 75 to 90% (4). The American Association for the Study of Liver Diseases (AASLD) practice guidelines recommend the following treatment regimens as first-line therapies (4): the initial treatment with prednisolone (PRED) 60 mg/day or a combination of PRED 30 mg/day and azathioprine (AZA) 50 mg/day. PRED treatment may lead to a poor response and steroid-related adverse effects. These adverse effects depend on the dose and time, occurring with the use of doses over 7.5-10 mg/day taken for more than a few months (4). The most common adverse effects are Cushingoid findings, while nearly half of the patients had their treatment discontinued due to cosmetic reasons and obesity (5). Other adverse effects of steroid use are rare and include osteoporosis, diabetes, cataracts, psychosis, myopathy, and hypertension (4). The majority of adverse effects occurs due to high-doses in the initial treatment regimens and are often reversible (6-8). Therefore, alternative treatments have been used in the AIH, such as cyclosporine-A, tacrolimus, mycophenolate mofetil, budesonide (BUD), and ursodeoxycholic acid (9-11). Data on the efficacy and adverse effects of BUD in the first line treatment of AIH are limited. Therefore, we aimed to compare the early treatment efficacy and adverse effects of patients receiving BUD and PRED in the first-line treatment of AIH.

This retrospective study compared patients receiving PRED/AZA treatment and those receiving BUD/AZA treatment in terms of demographic data, liver biopsy activity scores, laboratory findings at baseline and after six months of treatment, and the rates of adverse effects.

## Material and methods

### Patient groups and study design

The study involved the last 25 patients treated with BUD/AZA and the last 25 patients treated with PRED/AZA in the first line treatment of AIH at the Tepecik Training and Research Hospital who were 18-year old or older and diagnosed with definite AIH according to the revised diagnostic criteria (score >15) by the International Autoimmune Hepatitis Group (9) between February 2015 and February 2018. The patients were treated with BUD/AZA because of comorbid diseases (obesity, hypertension, brittle diabetes, postmenopause, glaucoma). The included patients were required not to have received previous AIH treatment and had serum aspartate aminotransferase (AST) and alanine aminotransferase (ALT) levels two times higher than the reference levels. Exclusion criteria were viral infections including hepatitis A, B, C, D, and E; chronic liver diseases such as primary biliary cirrhosis, primary sclerosing cholangitis, Wilson's disease, or hemochromatosis; and liver cirrhosis and alcohol consumption.

In the PRED/AZA group, the treatment was started with a low dose of 30 mg/day PRED and 50 mg/day AZA. PRED was reduced to 10 mg/day according to weekly follow-up at the end of first month, and this dose was continued as the maintenance therapy. In the BUD/AZA group, the treatment was started with 9 mg/day BUD and 50 mg/day AZA. BUD was continued with 9 mg/day as the maintenance therapy. In both groups, AZA was increased to 1.5 mg/kg/d in the first month with weekly follow-up.

Patients’ demographic data, autoimmune antibodies levels, liver biopsy results (necroinflammatory activity and fibrosis scores) were compared between the groups at baseline. The levels of AST, ALT, alkaline phosphatase, gamma-glutamyl transferase, total bilirubin, direct bilirubin, hemoglobin, white blood cell, platelet, prothrombin time, and the international normalized ratio were compared between baseline and 6th month. ANA, SMA, anti-LKM1, and anti-LC1 were evaluated by indirect immunofluorescence on snap-frozen sections of the rat liver, kidney, and stomach. Necroinflammation activity and fibrosis stage were assessed by the Ishak modified histology activity index grading and staging system (12).

The groups were compared after six months of treatment with regards to the rates of complete biochemical response (AST and ALT levels reaching the reference levels), adverse effects (moon face, acne, hirsutism, striae, and buffalo hump; myopathy, etc), and treatment discontinuation. The study was approved by the Ethics Committee of Tepecik Training and Research Hospital (No: 2019/5-3).

### Statistical analysis

The Kolmogorov-Smirnov test was used for normality testing. Numerical variables are expressed as medians and interquartile ranges (IQR). χ^2^ test was used for the comparison of categorical values between the groups. Mann-Whitney U test was used for the comparison of continuous independent variables, while Wilcoxon signed rank test was used for the comparison of dependent variables. The level of statistical significance was set at *P* < 0.05. Statistical analyses were performed with SPSS 22.0 (IBM, Armonk, NY, USA).

## Results

### Baseline results

The majority of study participants were women (40 out of 50), which is in accordance with the literature (1,13), and 94% of the patients had autoantibody positivity. The groups did not significantly differ in sex, mean age, autoantibody positivity, baseline necroinflammatory activity scores, and fibrosis scores (Table 1).

**Table 1:** Age distribution, immunoglobulin G levels, necroinflammation, and fibrosis scores of patients receiving prednisolone/azathioprine treatment and those receiving budesonide/azathioprine treatment

	Total	Prednisolone/azathioprine	Budesonide/azathioprine	P*
	Median (IQR)	Median (IQR)	Median (IQR)	
Age (year)	47 (21)	48 (19.5)	43 (23.5)	>0.999
Immunoglobulin G (mg/dL)	1975 (310)	1960 (235)	2010 (415)	0.356
Necroinflammation score (reference range: 0-18)	8 (5.3)	7 (5)	8 (6)	0.089
Fibrosis score (reference range: 0-6)	3 (3)	2 (3)	3 (2)	0.14

*Mann-Whitney U Test.

### Biochemical laboratory findings

The groups at baseline and after six months of treatment did not significantly differ in laboratory findings other than the IgG levels (Table 2). IgG levels were not compared due to missing data in 30 patients.

**Table 2:** Comparison of laboratory findings of patients receiving prednisolone/azathioprine treatment and those receiving budesonide/azathioprine treatment at baseline and after six months of treatment*

	Total	Prednisolone/azathioprine	Budesonide/azathioprine	*P^†^*
	Median (IQR)	Median (IQR)	Median (IQR)	
AST (U/L)				
baseline	99 (150.3)	89 (127.5)	123 (232.5)	0.322
6th month	31.5 (14.5)	32 (12)	31 (19.5)	0.877
*P^‡^*	<0.001	<0.001	<0.001	
ALT (U/L)				
baseline	110 (128.8)	107 (101.5)	132 (212)	0.393
6th month	32 (18)	32 (10.5)	26 (24)	0.207
*P^‡^*	<0.001	<0.001	<0.001	
Total bilirubin (mg/dL)				
baseline	0.8 (0.5)	0.7 (0.4)	1 (0.5)	0.385
6th month	0.7 (0.2)	0.7 (0.3)	0.7 (0.4)	0.724
*P^‡^*	<0.001	0.006	0.009	
Direct bilirubin (mg/dL)				
baseline	0.3 (0.5)	0.2 (0.5)	0.3 (0.5)	0.854
6th month	0.5 (0.3)	0.5 (0.2)	0.5 (0.4)	0.815
*P^‡^*	0.028	0.277	0.045	
ALP (U/L)				
baseline	136 (95.8)	137 (101)	136 (157)	0.567
6th month	85.5 (52.3)	76 (52.5)	89 (47.5)	0.317
*P^‡^*	<0.001	<0.001	<0.001	
GGT (U/L)				
baseline	85.5 (111.3)	76 (77)	90 (188)	0.229
6th month	32 (32)	32 (52)	31 (31)	0.449
*P^‡^*	<0.001	<0.001	<0.001	
Albumin (g/dL)				
baseline	4.1 (0.4)	4.2 (0.4)	4.1 (0.5)	0.063
6th month	4.2 (0.2)	4.2 (0.2)	4.2 (0.3)	0.142
*P^‡^*	0.011	0.461	0.003	
WBC (/μL)				
baseline	6450 (1,983)	6900 (2,155)	6300 (1,900)	0.351
6th month	7750 (2,425)	7800 (2,800)	7700 (2,600)	0.808
*P^‡^*	0.010	0.179	0.032	
Hemoglobin (g/dL)				
baseline	12.5 (1.9)	12.3 (2.3)	12.7 (1.8)	0.308
6th month	12.7 (1.5)	12.3 (1.4)	13 (1.7)	0.190
*P^‡^*	0.135	0.331	0.229	
Platelet ( × 10^3^/μ)				
baseline	259.5 (139.3)	263 (140.5)	255 (126)	0.900
6th month	290 (122)	295 (155)	289 (91.5)	0.594
*P^‡^*	0.012	0.027	0.253	
PT (s)				
baseline	11.6 (2)	11.6 (2.2)	11.6 (2.2)	0.961
6th month	11 (1.5)	11.4 (1.5)	11 (1.7)	0.173
*P^‡^*	<0.001	0.062	0.001	
INR				
baseline	1.1 (0.2)	1.1 (0.2)	1 (0.1)	0.420
6th month	1 (0.2)	1 (0.1)	0.9 (0.2)	0.102
*P^‡^*	0.001	0.027	0.011	

*AST – aspartate aminotransferase; ALT – alanine aminotransferase; ALP – alkaline phosphatase; GGT – gamma-glutamyl transferase; WBC – white blood cell; PT – prothrombin time; INR – international normalized ratio.

†Between groups (Mann-Whitney U Test).

‡Within groups (Wilcoxon signed rank Test).

### Biochemical response and adverse effects

There was no significant differences between the groups in biochemical response rates (17 or 68% in PRED/AZA group vs 18 or 72% in BUD/AZA group, *P* = 0.231). There was also no significant difference in the rates of steroid-related adverse effects (9 or 36% of patients in PRED/AZA group and 5 or 20% of patients in BUD/AZA group during the 6-month follow-up, *P* = 0.173) (Table 3). The number of adverse effects in the PRED/AZA and BUD/AZA groups was 15 and 7, respectively. Two patients in the PRED/AZA group discontinued PRED due to severe myopathy and Cushingoid symptoms, and the treatment was continued as AZA monotherapy (Table 4). Three patients in both groups had the dose modified due to leukopenia. The treatment was not stopped or interrupted in any patient due to adverse effects related to AZA therapy.

**Table 3:** Biochemical complete response and adverse effect rates in treatment groups

	Prednisolone/azathioprine (n = 25)	Budesonide/azathioprine (n = 25)	P*
**Biochemical complete response, n (%)**	17 (68)	18 (72)	0.231
**Adverse effect, n (%)**	9 (36)	5 (20)	0.173

*χ^2^ test.

**Table 4:** Total adverse effects and treatment discontinuation rates among treatment groups

	Prednisolone/azathioprine (n = 25)	Budesonide/azathioprine (n = 25)
**Total adverse effects**	**15**	**7**
**Acne**	**4**	**2**
**Hirsutism**	**3**	**1**
**Myopathy**	**1**	**1**
**Moon face**	**4**	**1**
**Buffalo hump**	**1**	**1**
**Striae**	**2**	**1**
**Discontinued treatment**	**2**	**0**
**Myopathy**	**1**	**0**
**Hirsutism**	**1**	**0**

Received: February 12, 2019

Accepted: August 20, 2019

## Discussion

This study showed no differences in biochemical response between the groups and a lower, although not significantly, rate of adverse effects and lower total number of adverse effects in BUD/AZA group.

The purpose of AIH treatment is to improve the clinical symptoms, transaminase and IgG levels, and histological activity. While the reduction of transaminase levels below two times the upper reference limit was previously considered as remission, it has been recently established that disease can progress in the presence of abnormal transaminase levels (14). The treatment is reduced to a minimum immunosuppressive dose for maintenance once remission is achieved.

BUD is a synthetic glucocorticoid with potent topical effects, undergoing first-pass elimination in a healthy liver (>90%) (15). It is not recommended for treatment in patients with liver cirrhosis and associated systemic shunts because the first-pass elimination does not occur in these individuals and systemic effects are increased (16,17). BUD, which has been used in AIH for 20 years, is associated with fewer adverse effects and improves hepatic inflammatory activity (11,18). There are conflicting literature data regarding the combination of BUD and AZA. A prospective, randomized trial of 203 naive or relapsed patients excluding cirrhotic patients showed that more patients had a complete biochemical response in the BUD arm than in the PRED arm (19). A retrospective study of 60 patients showed that the biochemical response rate at the end of a six-month period in patients who were switched from PRED to BUD (owing to unresponsiveness or adverse effects) was 55% (20). Twenty-five percent of the patients were switched back to PRED treatment due to BUD-related adverse effects or an insufficient response (20). However, remission was maintained in all patients who had been already in remission at the time of medication switch. BUD treatment may be an alternative treatment option to standard PRED treatment in children (21); however, Czaja and Lindor (22) reported lower remission rates in patients receiving BUD. Danielsson and Prytz (18) reported that BUD treatment reduced liver inflammation only in patients with non-cirrhotic AIH. Furthermore, Csepregi et al (23) reported that a complete response was achieved in 83% patients receiving BUD at the 6th month and cirrhosis was observed in 2 of 3 patients who could not respond to BUD treatment. In our study, the biochemical response rate at the 6th month in patients receiving BUD/AZA treatment was 72%, which is lower than in the study by Csepregi et al (23) but higher than in the study by Manns and Werner et al (19,20).

The most common problem in patients receiving long-term steroid treatment are steroid-related adverse effects. Although their incidence was reduced with the introduction of immunosuppressive drugs such as AZA, they were reported in 44%-69% of patients receiving steroid therapy alone and in 10%-47% of patients receiving an AZA combination regimen (4,6,16). In our study, steroid-related adverse effects were reported in 9 (36%) patients in the PRED/AZA group and in 5 (20%) patients in the BUD/AZA group. The total number of adverse effects in the PRED/AZA and BUD/AZA groups was 15 and 7, respectively. The treatment was discontinued in 2 (8%) patients receiving PRED/AZA therapy owing to severe myopathy and hirsutism, while no serious adverse effects leading to treatment discontinuation were observed in the patients receiving BUD/AZA treatment.

Although the duration of immunosuppressive therapy has not been definitely established, practice guidelines of both the AASLD and the European Association of Liver Research recommend that the treatment should be continued for at least 2 or 3 years, and that biochemical remission should be maintained for 2 years before discontinuation. The practice guidelines also recommend liver biopsy before treatment discontinuation since histological inflammatory activity may persist despite biochemical remission (4,17). It should be noted that 80% of the patients whose treatment was discontinued experienced a relapse within three years, and the majority of patients may require a lifelong treatment (24). These issues stress the need for treatment modalities with fewer adverse effects and higher efficacy. Based on our findings, we can argue that BUD/AZA treatment is as efficacious as PRED/AZA treatment in achieving an early biochemical response but that it may cause fewer adverse effects.

However, these results have to be viewed in light of some limitations. The study had a retrospective design and limited number of patients, and did not include data on long-term response rates. In addition, no data on liver necroinflammatory activity and fibrosis scores at the 6th month were available.

This study suggests that BUD is as effective as PRED in achieving remission in AIH, and we expect that it may become the first-line treatment of choice, especially in patients with obesity, diabetes, treatment-resistant hypertension or glaucoma owing to the lower adverse effects rates. Naturally, these findings should be supported by further prospective studies investigating liver histology.
